# Histological and Histomorphometrical Evaluation of a New Implant Macrogeometry. A Sheep Study

**DOI:** 10.3390/ijerph17103477

**Published:** 2020-05-16

**Authors:** Sergio Alexandre Gehrke, Margherita Tumedei, Jaime Aramburú Júnior, Tiago Luis Eirles Treichel, Roni Kolerman, Stefania Lepore, Adriano Piattelli, Giovanna Iezzi

**Affiliations:** 1 Department of Research, Biotecnos, Cuareim 1483, Montevideo CP 11100, Uruguay; sergio.gehrke@hotmail.com (S.A.G.); jaimearamburujunior@gmail.com (J.A.J.); 2 Department of Biotechnology, Universidad Católica de Murcia (UCAM), 30107 Murcia, Spain; 3 Department of Medical, Oral and Biotechnological Sciences, University of Chieti, 66100 Chieti, Italy; lepore.s@libero.it (S.L.); apiattelli@unich.it (A.P.); gio.iezzi@unich.it (G.I.); 4 Department of Surgery, Faculty of Medicine Veterinary, University of Rio Verde, Rio Verde 75900-000, Brazil; tiago@unirv.edu.br; 5 Department of Periodontology and Dental Implantology, The Maurice and Gabriela Goldschleger School of Dental Medicine, Tel Aviv University, Tel Aviv 6997801, Israel; kolerman@netvision.net.il

**Keywords:** dental implants, healing chamber, osseointegration, osteogenic matrix

## Abstract

Decompression or healing chambers between the threads have been proposed to improve and accelerate the osseointegration process of dental implants. The aim of the present work was to test, in an in vivo sheep study, if healing chambers between the threads could produce a better osseointegration process. Thirty titanium implants (15 conventional design (control) and 15 implants with healing chambers (test)) were inserted in a random fashion in the tibia of 3 sheep. The animals were euthanized after 30 days of healing, and the retrieved specimens treated to obtain thin ground sections. Histological observations showed that the quantity of newly formed bone growing in an apical direction was lower in the control group (1095 µm) when compared to the Test group (1658 µm). This difference was statistically significant. Moreover, a layer of osteogenic matrix was present around the portion of implants immersed in the marrow spaces. This osteogenic tissue was thicker in the test group. In conclusion, the present study confirmed the very good results in implants with healing chambers that presented a higher percentage of new bone formation.

## 1. Introduction

The osseointegration of implants involves a cascade of biological events at the cellular and extracellular levels, on the interface of bone tissue and the implant surface, seeking to ensure that this surface is covered with a newly formed bone [1]. Among the biological events, we can include the activation of osteogenic processes, which are similar to the processes of bone fracture repair [2,3]. In addition, the cascade of biological events is regulated by the growth and differentiation factors released at that location [4]. After osseointegration of the implant, when it is put into function under physiological conditions, the extent of the loads and tensions will be discharged into the supporting bone structures. This transfer of occlusal forces to the bone-implant interface is a fundamental factor in determining the result of long-term implant treatment, and a material design capable of distributing the functional forces to the supporting structures within physiological values is essential [5,6].

During implant insertion in the bone site, there is always a strain in the surrounding bone [1]. This strain can be tolerated by the osseous tissues up to a threshold, due to a capability of the bone to undergo relaxation [7]. If and when this capacity is exceeded, there is the possibility of the production of bone microfractures and vessel compression with a possible traumatic or ischemic necrosis of the osseous tissue [7,8,9,10]. This latter fact could, on the other hand, produce a rapid resorption of the peri-implant bone tissue [7]. In the last decade, several paper have reported that, in press-fit conditions during implant insertion, no space was present between implant and bone, while, on the contrary, when there were lower degrees of compression of the peri-implant bone, a higher amount of bone formation was observed [7,8,9,10]. The macrogeometry of the implant is then considered a factor of great importance in the process of osseointegration [8,9,11,12], and expectations of an improved bone healing via implant macrostructure modifications have been reported [13]. An ideal implant shape should provide a balance between compressive and tensile forces. Papers have presented data on retrieved human implant where a free space, called the healing chamber, had been created between the implant threads [10,11,14]. Lamellar bone with a Haversian-like canal system was present in these healing chambers [10,14]. A new implant has been designed with decompression chambers between the threads to improve and accelerate the osseointegration process compared with a conventional implant design [9,15]. This new implant design has been tested in a rabbit study [15] where a significant enhancement of the osseointegration processes were found, with an increase in the bone-to-implant contact and in the bone area fraction inside the threads. Moreover, Gehrke et al., in an in vitro study where implants with this new design were inserted in polyurethane foam sheets, found that there was a decrease in the insertion torque values, without changes, however, in the implant stability values [9].

The study hypothesis of the present investigation was that the healing chambers between the threads could produce a bone decompression during implant insertion, in a larger animal (sheep), and subsequently a better osseointegration process.

## 2. Materials and Methods

### 2.1. Implants Preparation and Groups Formation

**Figure 1:**
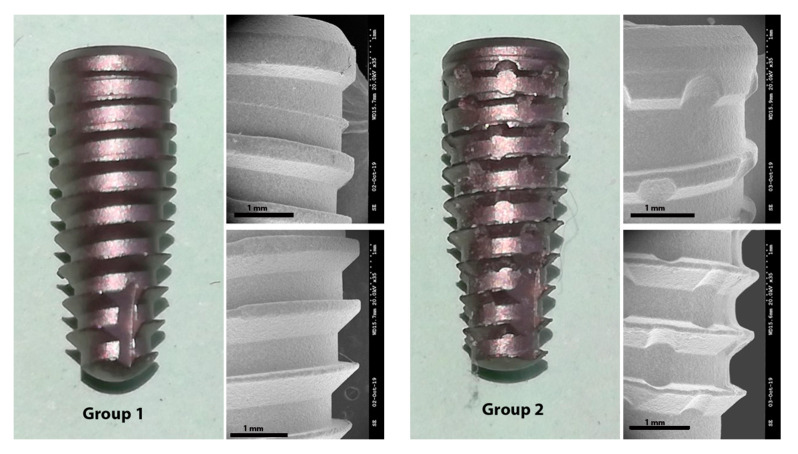
Representative image of the implants tested in each group. SEM images with magnification of ×35.

Thirty conical implants, 4 mm in diameter and 10 mm in length, with Morse taper connection were used. These implants were divided into two groups: Group 1 (control), with 15 implants Due Cone, that presented a conventional threads design; and, Group 2 (test), with 15 implants with the new macrogeometry (Maestro implant), that presented circular healing chambers (0.6 mm in diameter and 0.2 mm in depth) between the threads. Both implants have been manufactured by Implacil De Bortoli (São Paulo, Brazil) and are shown in Figure 1.

The implants of both groups were made by commercially pure titanium grade IV and, received a surface treatment by blasting with microparticles of titanium oxide, and a subsequent etching with maleic acid. The surface roughness of both groups showed an average for the Ra parameter of 0.56 ± 0.10 microns. All implant samples were subjected to washing, decontamination, sterilization, and packaging in accordance with current regulatory standards for this type of product.

### 2.2. Animal Experimentation

Three adult female Santa Ines sheep, weight between 35 and 40 kg, and age 2 ± 0.5 years were used. All animals were in good health conditions and were vaccinated against diseases pre-operatively and were screened to ensure good physical conditions (claws and worms). The animals were kept in individual cages and received water and mineral salt ad libitum throughout the study. The supervision of animal care, diet and pre- and post-operative fasting were conducted by a veterinary responsible for the sheep of the Veterinary School of the University of Rio Verde (Brazil). The animal experiment and breeding were performed under conditions approved by the Ethical Animal Committee of the University of Rio Verde (CEP/UnRV #18/2018). All titanium implants (15 per group) were implanted in sheep tibia (5 per tibia). The insertion of all implants (Group 1 and Group 2) was made in a random way using a website (www.randomization.com). All implants were distributed in a more central area of the tibia, 7 cm away from each articulation. The insertion torque was 30 ± 2 N for group 1 implants and 20 ± 2 N for group 2 implants, measured with a manual torquimeter. All implants have been inserted manually.

**Figure 2:**
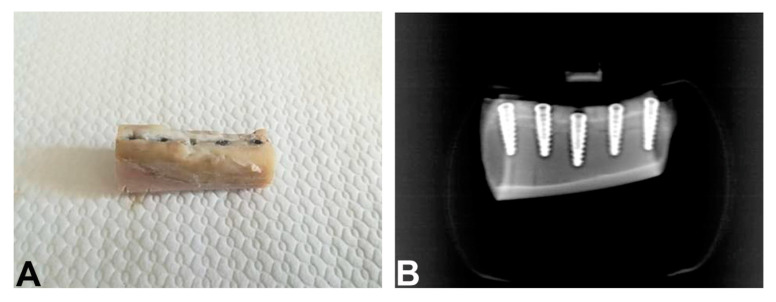
Block section of tibia (**A**). Rx of the implants inserted in tibia after 1 month of healing (**B**).

Before surgery, food and water were withheld for 24 h. All procedures that could result in anxiety and/or pain for the animals were conducted under anesthesia. For the intramuscular pre-anesthesia were used 0.3 mg/kg of Midazolam (Pfeizer Brasil Ltd.a., São Paulo, Brazil) plus 2 mg/kg of Tramadol (Laboratório Teuto Brasileiro S/A, Anápolis, Brazil). After 20 min, when the animals were visibly sedated and exhibited no responses to pain, cephalic vein cannulation was performed and lactated Ringer’s solution (5 mL/kg/h/i.v.; Baxter Hospitalar Ltd.a, São Paulo, SP, Brazil) was infused. Anesthesia was then induced with propofol (4 mg/kg/i.v., Diprivan^®^; Astra Zeneca, Cotia, SP, Brazil). The animals were intubated (orotracheal intubation), and the general anesthesia was maintained by inhalation of 1% isoflurane (Isoforine^®^; Cristália, Itapira, Brazil). After the start of anesthesia, lidocaine (4 mg/kg, Xylestesin^®^; Cristália, Itapira, Brazil) and morphine (0.1 mg/kg, Dimorf^®^; Cristália, Itapira, Brazil) were used for epidural block. The anesthetic procedures were performed under veterinary supervision. All surgical procedures were performed under sterile conditions, the surgical area was shaved, washed, and disinfected with iodo-povidone at 10%. The bone surface in the tibia region was exposed by an incision followed by a separate elevation of skin and periosteum. Each site was perforated within a ~15 mm distance of the other. Under constant irrigation with 0.9% sodium saline solution, the perforations were performed with a surgical drill according to the manufacturer’s surgical protocol. All drilling procedures were conducted at 1200 rpm. All implants were installed in the tibia at 24 rpm. The periosteum around the bone perforations was placed back in position and attached to the subcutaneous tissue using an interrupted suture. All animals were euthanized 30 days after the implantations with an overdose of anesthetic. Block sections of tibia, containing the implants, were obtained, and all the specimens underwent an x-ray examination, to identify the longitudinal axis of the implants (Figure 2A,B).

### 2.3. Postoperative Care

Post-operative pain and inflammation were controlled with the administration of tramadol (2 mg/kg/i.v., Laboratório Teuto Brasileiro S/A, Anápolis, Brazil) and meloxicam (0.5/kg/oral, Meloxivet^®^; Duprat, Rio de Janeiro, RJ, Brazil) for 3 days. During the first post-operative week, antibiotic prophylaxis was administered using oxytetracycline (0.1 mg/kg/i.m., Terramicina^®^; Pfizer do Brasil, São Paulo, Brazil). Silver spray was topically applied daily to prevent local infection. After the surgery, the animals received diet and had free access to drinking water.

### 2.4. Histology

The biopsies were fixed by immediate immersion in 10% buffered formalin and processed (Precise 1 Automated System; Assing, Rome, Italy) to obtain thin ground sections, with a cutting-grinding system, as previously described [16]. The specimens were dehydrated in an ascending series of alcohol rinses and embedded in glycol-methacrylate resin (Technovit 7200 VLC; Kulzer, Wehrheim, Germany).

After polymerization, the specimens were sectioned, along their transversal axis, with a high precision diamond disk at about 150 µm and ground down to about 30 µm with a specially designed grinding machine Precise 1 Automated System [10]. Three slices were obtained from each specimen, subsequently stained with acid fuchsin and toluidine blue before the analysis. Histological analysis was carried out using a light microscope (Laborlux S, Leitz, Wetzlar, Germany) connected to a high-resolution video camera (3CCD, JVCKY-F55B, JVC, Yokohama, Japan) and interfaced with a monitor and PC (Intel Pentium III 1200 MMX, Intel, Santa Clara, CA, USA). This optical system was associated with a digitizing pad (Matrix Vision GmbH, Oppenweiler, Germany) and a histomorphometry software package with image capturing capabilities (Image-Pro Plus 4.5, Media Cybernetics Inc., Immagini & Computer Snc, Milano, Italy).

In all specimens, the measurements were made of the following landmarks: The top of the implant (TI); the first bone implant contact (FBIC); the lower portion of the cortical bone (LCB), the most apical newly formed bone in contact with the implant (NB); the apex of the implant (A). All measurements were made at both sides of the implant; in parallel to the long axis of it.

The linear distances between TI and FBIC, FBIC and LCB, LCB and NB, NB, and A, were measured parallel to the long axis of the implant at both sides of the implant at a magnification of ×100. Moreover, the amount of new bone, old bone, soft tissues (marrow spaces, osteogenic matrix), and other tissues in contact with the implant surface were measured at a magnification of ×200.

The first bone implant contact was measured between TI and FBIC, while the newly formed bone, in contact with the implant surface, was measured between LCB and NB. Both the measurements were made at a magnification of ×100.

**Figure 3:**
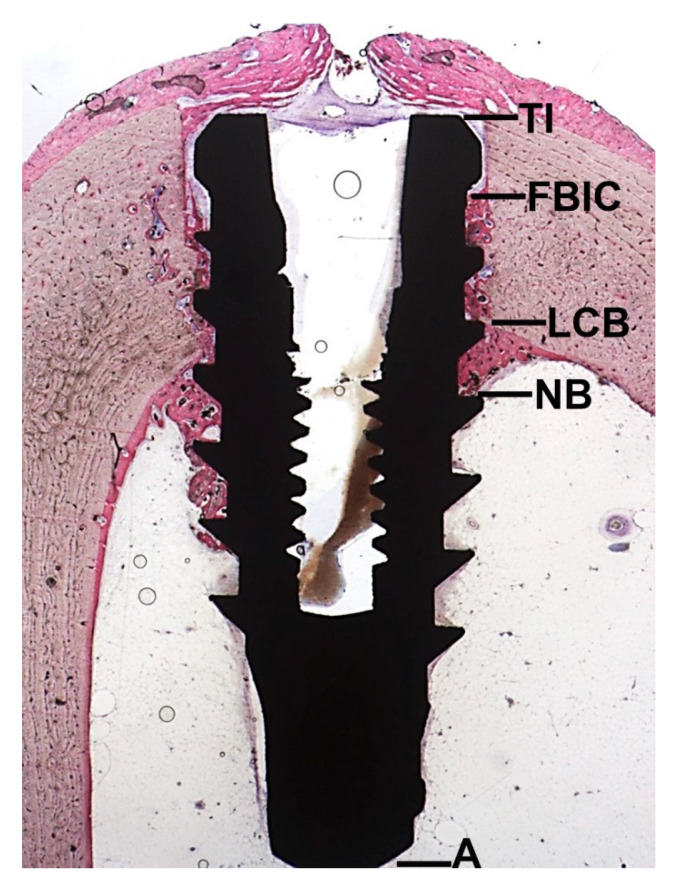
Histological image illustrating the landmarks for the histological evaluation. Top of the implant (TI); first bone implant contact (FBIC); lower portion of the cortical bone (LCB), most apical newly formed bone in contact with the implant (NB); apex of the implant (A).

The distance between FBIC and LCB (cortical compartment) and the distance between NB and A (medullar compartment) were also evaluated to measure all tissues in contact with the implant surface. Particularly, in the cortical space the presence and the amount of new bone, pre-existing bone and soft tissues were evaluated. In the marrow cavity, the newly bone formation and the amount of bone marrow and osteogenic matrix were measured. All the measurements were made at a magnification of ×200, in a blinded way, by GI and SL (Figure 3).

### 2.5. Statistical Analysis

Descriptive statistical analysis was evaluated by the mean values, standard deviation (SD) and lower-upper 95% confidence intervals (CI) of the tested variables. The –Smirnov test was used to evaluate the normal distribution of the study data. Differences between the experimental conditions were analyzed by Wilcoxon signed rank test included in the Prism 6 (GraphPad, San Diego, CA, USA). A *p* < 0.05 were considered statistically significant.

## 3. Results

**Figure 4:**
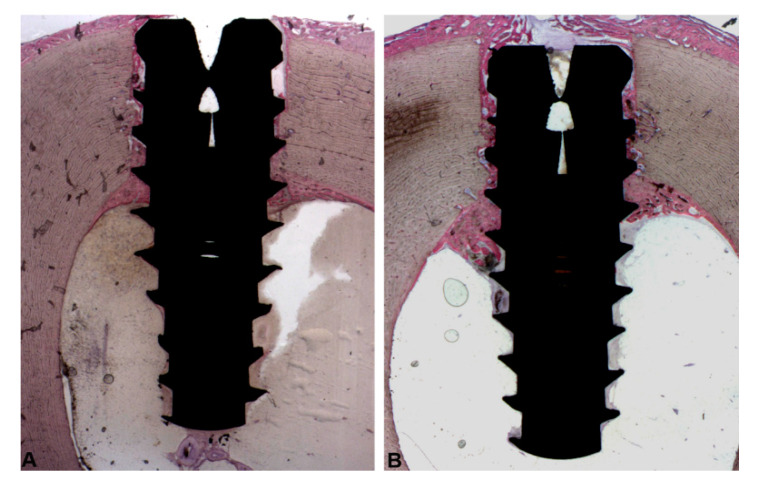
At low-magnification, healing of the tissues 30 day after implant placement in Group 1 (**A**) and Group 2 (**B**). The coronal portion of the implants was located in the cortical portion while the middle and apical implant portions were immersed in the marrow cavities of the tibiae. (Acid fuchsin-Toluidine blue 6X).

All implants were available for histological evaluation. However, 5 implants were not integrated (4 in the Group 1 and 1 in the Group 2) because a complete displacement of the implants in the marrow cavities of the tibia was observed. Then, a total of 11 implants for the Group 1, and 14 implants for the Group 2 underwent analysis. At low magnification, in all samples it was possible to observe that the coronal portion of the implants were in contact with cortical bone while the middle and apical implant portions were included in large marrow cavities, in both groups (Figure 4A,B). Histological analysis was performed both in the cortical and marrow portions.

### 3.1. Cortical Portion

**Figure 5:**
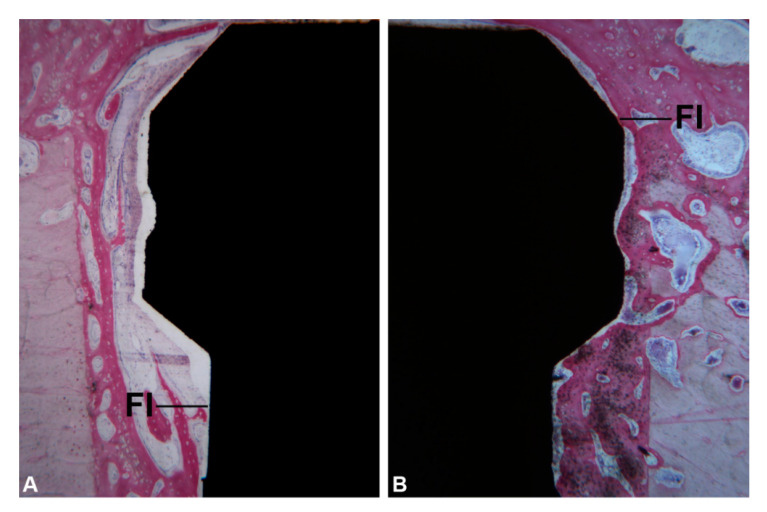
Cortical Compartment. These images showed the first bone implant contact (FI) in the coronal portion in Group 1 (**A**) and in Group 2 (**B**). New bone formation was observed in both groups, but in Group 2 it occurred more coronally than in the Group 1. (Acid fuchsin-Toluidine blue 18X).

**Figure 6:**
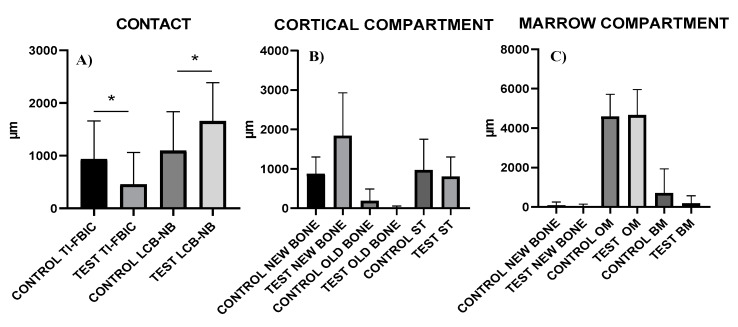
(**A**) Bar graph represents means and standard deviations of distribution of the Bone contact with the Implant surface; (**B**) Peri-implant tissues distribution at the level of the Cortical Compartment; (**C**) Peri-implant tissues distribution at the level of the Marrow Compartment (* *p* < 0.05).

After 30 days of healing, bone resorption and bone formation had occurred, and, therefore, the crestal bone was no longer at the same level of the implant shoulder as when the implants were inserted. In these portions it was possible to observe osteoblastic activity with a rim of osteoblasts, depositing osteoid matrix directly on the implant surface. The TI-FBIC was 932 ± 729 and 453.3 ± 605.3 µm for Group 1 and Group 2, respectively (Figure 5A,B) (Figure 6A–C, Table 1).

In this portion, a discrepancy between the host bone bed and the body of the implant explained why the pre-existing bone was present only in a minimal percentage both in the controls (192.1 ± 297.7 µm) and in the test (9.188 ± 48.62 µm) groups (Figure 6B and Table 2).

**Figure 7:**
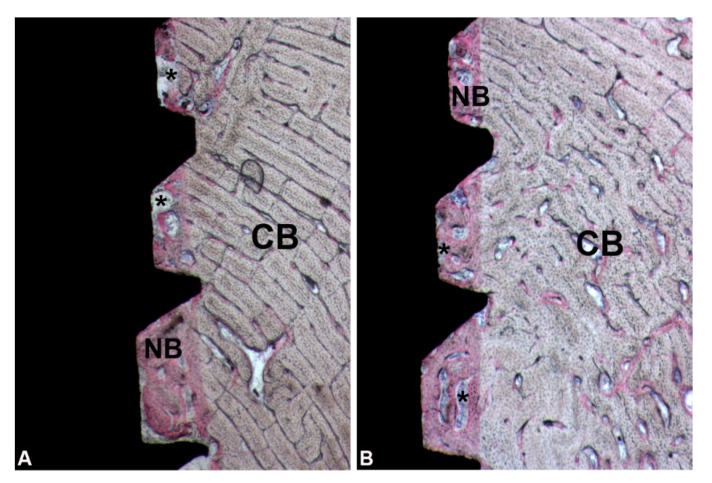
Cortical portion. New bone (NB) as well as small marrow spaces (*) between implant surface and cortical bone (CB) was observed in control (**A**) and in test (**B**) groups. (Acid fuchsin-Toluidine blue 18X).

Appositional bone healing was observed in the cortical component and the healing chambers between implant and bone were partially filled with woven bone. The contact between new formed bone and implant surface was 873.6 ± 428.3 µm in the control group, while in the test group was 1839 ± 1088 µm (Figure 6B and Table 2). In this portion, the soft tissues were poorly represented (Figure 7A,B).

### 3.2. Marrow Compartment

**Figure 8:**
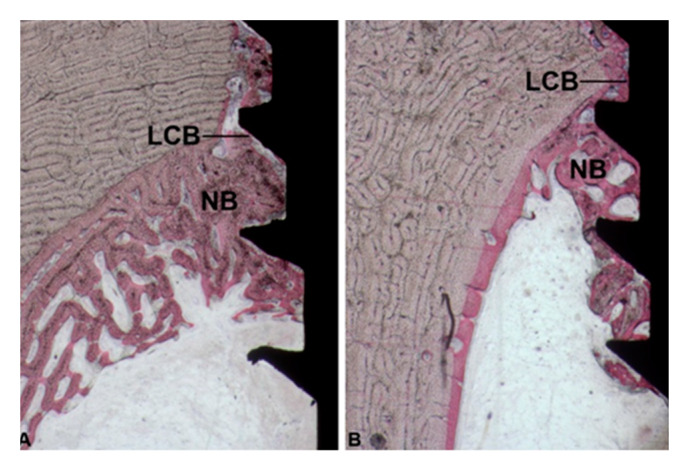
(**A**,**B**) Marrow portion. In the coronal portion new bone formation (NB) close to the implant surface was observed. It started from the pre-existing bone (LCB) and had grown on the implant surface in an apical direction. In the test group (**B**) more new bone formation (NB) was present. (Acid fuchsin-Toluidine blue 18X).

**Figure 9:**
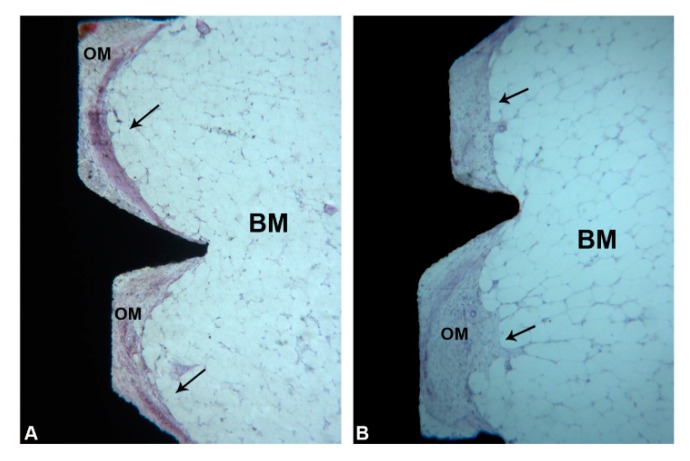
(**A**,**B**) Marrow portion. A layer of osteogenic matrix (OM) was observed close to the implant surface. This OM was clearly delimited from the bone marrow by a dense band (**black arrows**). (Acid fuchsin-Toluidine blue 18X).

**Figure 10:**
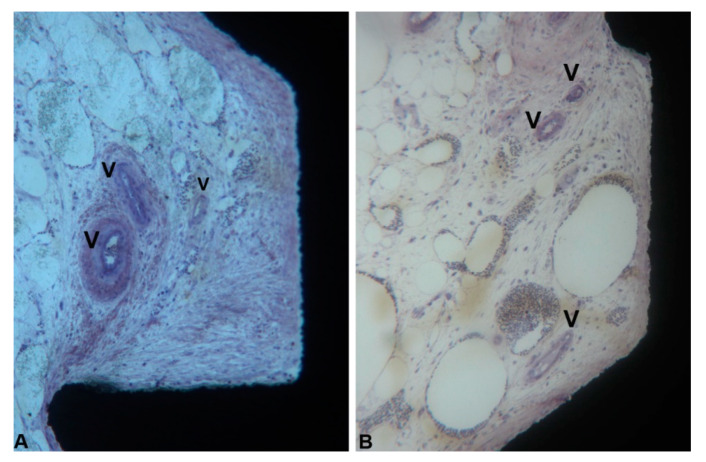
Marrow portion. Many blood vessels (V) were present near the implant surface in the concavities, both in group 1 (**A**) and group 2 (**B**). (Acid fuchsin-Toluidine blue 100X).

**Figure 11:**
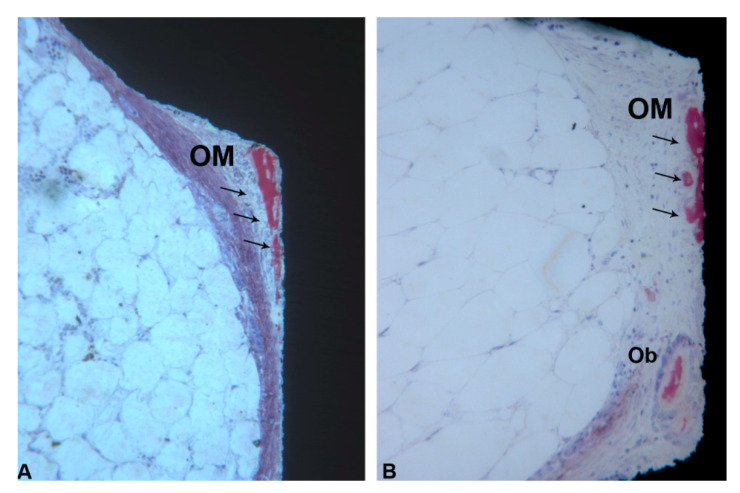
(**A**,**B**) Marrow portion. In the apical portion, a layer of osteogenic matrix (OM) inside the concavities was observed close to the implant surface and it was clearly delimited from the bone marrow by a dense band (**black arrows**) (Acid fuchsin-Toluidine blue 18X).

In the coronal part of the marrow compartment, near to the pre-existing bone, new bone formation close to the implant surface was observed. It started from the pre-existing bone (LCB) and had grown on the implant surface in an apical direction. In many areas, a few osteoblasts depositing osteoid matrix were present. The quantity of bone tissue was lower in the Control (1095 ± 743.1 µm) group compared to the Test (1658 ± 727.9 µm) group (Figure 8A,B) (Figure 6A and Table 1). This difference was statistically significant. In an apical direction, at the end of, and in continuation with the new-formed bone, a layer of osteogenic matrix was present. The thickness of this osteogenic matrix was from 14.4 to 185.6 µm in the Group 1 and from 15.1 to 270.9 µm in the Group 2 (Figure 9A,B). This osteogenic matrix was rich in stromal cells and blood vessels in both groups. No inflammatory cells were present in this portion (Figure 10A,B). In some cases, inside the concavities, where this osteogenic matrix was more represented, osteoblasts were seen in close connection with newly-formed bone, indicating an ongoing bone formation in direct contact with the implant surface; this bone formation occurred far from the pre-existing cortical bone. This osteogenic matrix was clearly delimited from the bone marrow by a dense band both in the control and test groups (Figure 11A,B). In the marrow compartment, the thickness of this osteogenic matrix was similar in both groups 4587 ± 1132 vs. 4660 ± 1294 µm (Figure 6C and Table 3).

## 4. Discussion

Initial, primary or mechanical stability is the result of the interlocking between bone tissue and implant at the interface [17], and the relationship between implant macrodesign/macrogeometry and surgical site size plays a key role in obtaining this type of stability [17,18]. The levels of the interfacial frictional forces are usually recorded by the insertion torque values [17]. Higher torque values lead to higher strains in the peri-implant bone with possible micro-cracks formation in the bone tissue, compression of the blood vessels, possible ischemic necrosis with subsequent resorption and remodeling of the perimplant bone [17]. In a rabbit study, the use of a, probably, too traumatic technique led to the formation of a 200–500 micron area of necrosis [18]. A way to decrease the levels of these forces at the interface could be an implant site with the same diameter of the external portion of the implant [13,17,19,20]. A consequence of this technique could be, however, a decrease of the primary stability [9]. In this latter technique, the insertion of the implant will be performed by tapping and not by screwing, with the formation of so-called healing chambers that are produced between the metal surface of the implant and the bone walls of the osteotomy site [20]. These healing chambers are then constituted by the empty spaces in the areas with no initial contacts between the metal surface of the implant and the external part of the neighboring peri-implant bone, immediately after implant insertion [21,22]. Wound or healing chambers have been introduced, in the last two decades, in the macrodesign of a few implant types [21,22,23,24]. Inside these healing chambers there is, first of all, the formation of a blood clot, characterized by the presence of many red blood cells immersed in a fibrin network [23] that progresses to the formation of an osteogenic stroma, rich in blood vessels [23,25], into which the osteogenic cells could migrate [13,17,19,20] to produce bone via an intramembranous-like path [21]. Moreover, in many cases, it will be possible to see a nucleation of bone throughout the healing chambers [17]. These healing chambers have been reported to be able to create an environment conductive to early bone formation at the interface [15,22].

Some further considerations must be done about the present results. Diameter dimension of the marrow cavity of the tibia is approximately 11–12 mm. This dimension has to be considered as a critical size defect. Regarding the osteoconductive process which contributes to the peri-implant bone healing, bone formation started from the pre-existing bone and went towards the implant surface. This concept was explained in Figure 3 where it was possible to find newly formed trabecular bone starting from the lower portion of the cortical bone (LCB). Newly-formed bone, found between the landmarks LBC and NB (apical newly formed bone in contact with the implant) showed the osteoconductive properties of the implant surfaces and it was the only portion where newly formed trabecular bone was observed within the marrow cavity.

In the histological results, it has been reported that stromal cells were present. Indeed, stromal cells are always present in the bone marrow. Their presence is a histological feature of the marrow cavity of the tibia, where the middle and apical portions of the implants were placed. In this portion (NB-A) the soft tissues in contact with the implant showed a higher density, while few spindle cells, typical of connective tissue, and many blood vessels were present. In the marrow cavity small areas of new bone formation were observed only in contact with the implant surface, while spicules of newly formed bone were not found in the areas distant from the metal surface. For this reason, in the present article, an important role has been attributed to the dense tissue in close contact with the implant, which was mainly present inside the threads [26,27]. There could be a role of fragments of bone transferred away from the cortex during the surgery. This hypothesis, could, however, probably be discarded because these fragments would have been easily recognizable from a histological point of view because they would have presented signs of remodeling that, on the contrary, were absent in the newly-formed bone found on the implant surface. Moreover, it must be considered that, in case of the presence of bone fragments displaced during the surgical insertion of the implants, this bone should have been visible also at a distance from the implant surface.

The two most relevant data obtained from the present investigation were:

- the statistically significantly higher percentage of newly formed formed bone in the implants where the healing chambers were present.
- a larger area, in the implants provided with healing chambers, of an osteogenic stroma, rich in blood vessels, where small, thin newly-formed bone trabeculae could be found, in close and tight contact with the implant portion immersed in the marrow compartment.

Gehrke et al. have shown that there was an increased bone density around implants provided with healing chambers [9], and Botticelli et al. [28], in implants inserted in sheep tibia, reported a bone formation in an apical direction at 2–4 weeks of healing.

The dense implant-attached connective tissue, characteristic of a provisional connective tissue, was: continuous with the primary spongiosa, about 50 microns wide, rich in collagen fibers, and, probably, should be regarded as an osteoid which, during continued modeling, will transform into woven bone, and establish bone-to-implant contact [29].

## 5. Conclusions

In conclusion, the present study confirmed the very good results in implants with healing chambers, with a significantly higher percentage of new bone formation and a larger area of a highly cellular and highly vascularized osteogenic matrix within the marrow spaces [30,31,32,33,34]. In this osteogenix matrix, newly formed bone spicules were found [33].

## Figures and Tables

**Table 1:** Bone tissue in contact with the implant surface, in the cortical portion.

Contact (μm)	Control Ti-Fbic	Test Ti-Fbic	Control Lcb-Nb	Test Lcb-Nb
Minimum	0.000	0.000	0.000	537.0
Maximum	2310	2377	2640	4116
Range	2310	2377	2640	3579
Mean	932.0	453.3	1095	1658
Std. Deviation	729.7	605.3	743.1	727.9
Std. Error of Mean	155.6	114.4	158.4	137.6

**Table 2:** Tissues quality at the level of the implant surface in the cortical portion.

Cortical Compartment (μm)	New Bone		Old Bone		Soft Tissue	
	Control	Test	Control	Test	Control	Test
Minimum	302,0	179.0	0.000	0.000	0.000	0.000
Maximum	1949	4857	979.1	257.3	3351	1746
Range	1647	4678	979.1	257.3	3351	1746
Mean	873.6	1839	192.1	9.188	969.0	805.2
Std. Deviation	428.3	1088	297.7	48.62	782.5	497.7
Lower 95% CI of mean	302.0	179.0	0,000	0.000	0.000	0.000
Upper 95% CI of mean	1949	4857	979.1	257.3	3351	1746

**Table 3:** Tissues quality at the level of the implant surface in the marrow compartment.

Marrow Compartment (μm)	New Bone		Osteogenic Matrix		Bone Marrow	
	Control	Test	Control	Test	Control	Test
Minimum	0.000	0.000	1612	1458	0,000	0.000
Maximum	715.9	316.4	6557	6823	4926	1588
Range	715.9	316.4	4944	5366	4926	1588
Mean	74.55	42.92	4587	4660	700,1	187.0
Std. Deviation	177.4	97.53	1132	1294	1233	375.7
Lower 95% CI of mean	−4.108	5.100	4085	4158	153,4	41.30
Upper 95% CI of mean	153.2	80.74	5089	5162	1247	332.6
